# A Comparative Study on Phenotypic versus ITS-Based Molecular Identification of Dermatophytes Isolated in Dakar, Senegal

**DOI:** 10.1155/2019/6754058

**Published:** 2019-12-18

**Authors:** Khadim Diongue, Ludivine Bréchard, Mamadou Alpha Diallo, Mame Cheikh Seck, Mouhamadou Ndiaye, Aida Sadikh Badiane, Stéphane Ranque, Daouda Ndiaye

**Affiliations:** ^1^Laboratoire de Parasitologie-Mycologie, CHU Aristide Le Dantec, BP 5005, Dakar, Senegal; ^2^Service de Parasitologie-Mycologie, Faculté de Médecine, de Pharmacie et d'Odontologie, Université Cheikh Anta Diop, BP 16477, Dakar, Senegal; ^3^Institut Hospitalo-Universitaire Méditerranée Infection, 13 005 Marseille, France

## Abstract

Classically, dermatophytes are identified by phenotypic methods even if these methods, sometimes, remain difficult or uncertain. On the other hand, nucleotide sequence analysis of internal transcribed spacers (ITS) of rDNA has proved to be a useful method for identification of dermatophytes. The objective of this study was to compare the phenotypic method with DNA sequencing of the ITS regions for identification of dermatophyte species isolated in Dakar, Senegal. A collection of thirty-two strains of dermatophytes were isolated from patients suffering from dermatophytosis. Mycological identification revealed *Trichophyton soudanense* (*n* = 13), *T. interdigitale* (*n* = 10), *Microsporum audouinii* (*n* = 5), and one strain for each of the following species: *T. rubrum*, *T. mentagrophytes*, and *M. canis* and one unidentified strain. For comparison, ITS-based PCR and DNA sequencing were applied for identification of the isolated dermatophytes. ITS sequences showed, in BLAST search analysis, 99-100% of similarity. Identification of dermatophyte isolates by conventional methods was confirmed by DNA sequencing of the ITS regions in 84% of cases. Discrepancies concern mostly *T. rubrum* misidentified as *T. interdigitale*. PCR sequencing provided an excellent tool for identifying dermatophyte strains that do not present typical morphological characteristics. It was also able to give correct identification of an atypical strain of *M. audouinii* responsible of mycetoma of the scalp.

## 1. Introduction

Dermatophytoses are relatively common and minor fungal infections, which are apparently evenly neglected over the African continent [1]. Dermatophytes are responsible for different clinical manifestations ranging from superficial as tinea corporis, tinea capitis, tinea pedis, and tinea unguium to subcutaneous affection such as dermatophytic mycetoma (pseudomycetoma) [2].

Particularly, tinea capitis requires specific treatment [3, 4]. Gräser et al. cited several reasons why identification of individual dermatophyte species causing infection remains important [5]. First, they gave the epidemiological circumstances promoting reinfection. For example, *Microsporum canis* commonly indicates an animal source, while *Nannizzia gypsea* (*M. gypseum*), causing similar lesions, indicates contact with contaminated soil. This identification of the infection source is important for a better management. Second, they invoked the actual treatment regimens which may differ for different dermatophyte species; for example, *Trichophyton tonsurans* in tinea capitis tends to require shorter treatment times than *M. canis* which to some extent evades drug exposure by forming arthroconidia outside the hair shaft. Third, distinction of dermatophytes from nondermatophytic species that do not respond to antidermatophyte therapy and causing dermatophytosis-like infection was pointed especially in onychomycosis. The classic example is dermatophytosis-like infection by *Neoscytalidium dimidiatum*. Likewise, dermatophytes must be distinguished from nonpathogenic fungi superficially resembling dermatophytes, such as *T. terrestre*, *Aphanoascus fulvescens*, and *Myriodontium keratinophilum*, regularly growing from dermatophytic lesions. Currently, terbinafine resistance of *Trichophyton* clinical isolates is increasingly reported from Asia, particularly in India [6]. Hence, the importance of dermatophytes identification to the species level may be useful.

Dermatophyte isolates can be identified to genus/species by phenotypic methods, based on colonial appearance, microscopic examinations, and biochemical tests such as growth patterns on *Trichophyton* agars and urease [3]. Phenotypic methods can be accurate when performed by skilled technicians, using standardized benchmarks to recognize and identify the exact features of the species [7].

Morphological identification of dermatophyte species in cultures is sometimes difficult or uncertain because there are variations from one isolate to another and overlapping characters between species [4]. That is why it is necessary to develop more reliable methods for identification of dermatophytes [7].

Increasingly, molecular biology techniques are adapted to the identification of dermatophytes and are more effective than conventional tests [8]. Several techniques for analyzing the genome of dermatophytes have been proposed, and DNA barcoding is very useful for accurate determination. The polymorphism of the internal transcribed spacers (ITS1 and ITS2) flanking the DNA sequence encoding the 5.8S rDNA is very sensitive and reliable for distinguishing different species of dermatophytes [4, 8].

This study aimed to compare the results of phenotypic and molecular identification by sequence analysis of a collection of clinical strains of dermatophytes isolated in Dakar, Senegal.

## 2. Materials and Methods

Between 2014 and 2017, strains were isolated from patients diagnosed with tinea capitis (*n* = 16, GenBank accession numbers from MN691044 to MN691059), interdigital tinea pedis (*n* = 9, GenBank accession numbers from MN691060 to MN691068), tinea unguium (*n* = 5, GenBank accession numbers from MN691069 to MN691073), tinea corporis (*n* = 1, GenBank accession number MN691074), and mycetoma of the scalp (*n* = 1, GenBank accession number MN691075) at the Parasitology and Mycology laboratory of Le Dantec University Hospital in Dakar, after dermatological consultation.

Diagnosis of dermatophytoses was done at the Parasitology and Mycology laboratory of Le Dantec University Hospital in Dakar, on the basis of mycological examination including direct and culture examination as described in a previous article [9]. Microscopic direct examination of all specimens was carried out in 20% KOH solution. All specimens were cultured in 2 plates/tubes, one containing Sabouraud dextrose agar (SDA) added with chloramphenicol (Bio-Rad, France) and the other containing SDA added with chloramphenicol and supplemented with cycloheximide (Bio-Rad, France). Cultures were incubated at 22–27°C and evaluated for growth after 48 h and then once weekly for a month.

Positive specimens with growth of dermatophytes were identified based on the growth rate and macroscopic and microscopic characteristics of colonies and sometimes on biochemical characteristics (urease test) [10, 11].

After morphological identification, colonies of dermatophytes were maintained by regular subcultures on SDA supplemented with cycloheximide.

### 2.1. DNA Extraction

DNA extraction was performed with the EZ1 Advanced XL instrument, Qiagen® from colonies isolated in 4 to 10 days culture at 25°C. A portion of colonies on culture medium was introduced into a bead tube containing 700 *μ*l of lysis buffer G2 (supplied with the EZ1 DNA Investigator kit, Qiagen®). This was followed by mechanical lysis with FastPrep®-24: 2 runs and by centrifugation at 10,000 rpm for 1 minute. Finally, 200 *μ*l of the supernatant was removed and placed in an EZ1 flat tube before launching the EZ1 Advanced XL instrument according to the instructions of the manufacturer. Genomic DNA was extracted, eluted in 200 *μ*l elution buffer, and stored at −20°C for downstream analyses.

### 2.2. DNA Sequencing

Molecular identification was performed by nucleotide sequence analysis of ITS of rDNA. The sequencing reactions were carried out with the same primers used for amplification. The latter was carried out with 5 *μ*L of DNA and 45 *μ*L of reaction volumes containing 25 *μ*L of AmpliTaq Gold® 360 Master Mix (Applied Biosystems®), 17 *μ*L of sterile water DNase/RNase free, and 1.5 *μ*L of each of forward and reverse primer. The PCR program was as follows: 94°C for 10 min, followed by 40 cycles of 94°C for 20 s and 53°C for 30 s and 72°C for 1 min, with a delay at 72°C for 7 min. Each sample was processed three times with the ITS1/2, ITS3/4, and ITS1/4 amplifying ITS1, ITS2, and ITS1-5.8S-ITS2 regions, respectively (Table 1) [12]. After revelation in 2% agarose gel, stained with ethidium bromide, and visualized under an UV illumination (E-gel® Imager), the best amplicon was used for sequencing. Purification of the amplified products was performed using Sephadex® G50 DNA purification, and the sequencing reactions were processed using a 3500 Genetic Analyzer (Applied Biosystems, Inc.). Obtained sequences were assembled using CodonCode Aligner software (CodonCode® Corporation).

### 2.3. DNA Analysis

ITS sequences were subjected to BLAST (Basic Local Alignment Search Tool) searches at GenBank (https://blast.ncbi.nlm.nih.gov/Blast.cgi) and MycoBank (http://www.mycobank.org). Identification from the both databases was retained when proposals were identical. Sequence-based species identification was defined by ≥99% sequence similarity.

## 3. Results

In total, we work with a collection of 32 dermatophyte strains.

**Figure 1:**
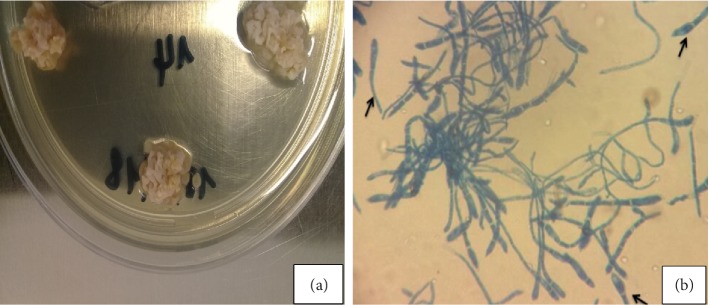
Macroscopic appearance showing raised and folded colonies (a) and microscoscopic aspect with “macroconidia” detaching from the hyphae (arrow) (b) of a dermatophyte isolated from mycetoma of the scalp unidentified by the morphological method and identified as *Microsporum audouinii* (GenBank accession number MN691075) by DNA sequencing of the ITS regions.

According to the phenotypic identification, isolates were identified as *T. soudanense* in 13 cases (40.6%), as *T. interdigitale* in 10 cases (31.3%), as *Microsporum audouinii* in 5 cases (15.6%), and in 1 case (3.1%) as *T. rubrum*, as well as for *T. mentagrophytes, M. canis*, and one unidentified strain (Figure 1).

ITS sequences showed 99-100% of BLAST similarity for the five species identified in our study.

The isolates were identified by DNA sequencing of the ITS regions, in 13 cases (40.6%) as *T. soudanense*, in 9 cases (28.1%) as *T. interdigitale*, in 6 cases (18.8%) as *M. audouinii*, in 3 cases (9.4%) as *T. rubrum*, and in 1 case (3.1%) as *M. canis*.

Thus, the identity of our strains was confirmed by DNA sequencing of the ITS regions at the species level in 27 of 32 cases (84.4%) and at the genus level in 31 of 32 cases (96.9%) (Table 2).

*T. soudanense and M. audouinii* were the most well-identified dermatophytes species by phenotypic method with proportions of 92.3% (12/13) and 83.3% (5/6).

The proportion of phenotypically identified cultures revealed by molecular study to have been misidentified was 15.6% (5/32).

The details of discrepancies between the phenotypic and molecular method are in asterisks in Table 2.

## 4. Discussion

In the present study, we sequenced the ITS region of rDNA of 32 dermatophyte strains isolated in Dakar from patients suffering from tinea capitis, tinea corporis, tinea pedis, tinea unguium, and mycetoma of the scalp. Our aim was to compare the obtained data with data generated using the phenotypic method to evaluate the accuracy of species identification of the isolates.

The phenotypic identification of our strains was confirmed by DNA sequencing of the ITS regions in 84.4% (27/32).

This result is in phase with that found by Li et al. with 81% (102/126) of correlation between phenotypic and ITS-based molecular identification of dermatophyte species, using a collection of clinical isolates [13].

On the other hand, other authors reported a better proportion with 94% of correlation between phenotypic and ITS-based molecular identification with a collection of 32 clinical isolates of dermatophytes [14].

Considering identification of the species included in this study, our findings show that *T. soudanense and M. audouinii* were the most well-identified dermatophyte species by the phenotypic method with proportions of 92.3% (12/13) and 83.3% (5/6), respectively.

This result could be explained by the fact that these species are the most commonly isolated dermatophytes in Senegal [15] and that mycologists are in the habit of identifying them.

One strain, unidentified by the phenotypic method was finally identified by DNA sequencing as *M. audouinii*. This atypical strain (GenBank accession number MN691075) produced grains in mycetoma. Microscopic examination of the grains, which were soft, on 20% potassium hydroxide mount, after washing in physiological saline and crushing between slide and coverslip, showed septate hyaline fungal hyphae on/in the grains. The scalp biopsy showed large grains composed of a compact mass of mycelium and vesicules, and a case report on this strain was published recently [2].

It has been recently emphasized that DNA sequencing, although a reliable technique for the identification of fungal species, could not properly distinguish between *T. rubrum*, *T. violaceum*, and *T. soudanense* [16]. These three latter fungi form the *T. rubrum* complex which is continually changing. According to Graser et al. in 2007 [7], the complex of anthropophilic dermatophyte species *T. rubrum* s.l. comprises two taxa, *T. rubrum* and *T. violaceum,* endemic to Africa and mainly causing tinea capitis and tinea corporis. Later, *T. soudanense* was re-established as a part of an “African population” of *T. rubrum* [5]. Then, more recently according to the revision of the dermatophyte species and the nomenclature of these fungi by de Hoog et al., *T. soudanense* is considered as a distinct species from *T. violaceum* [17]. Thus, *T. violaceum*, *T. soudanense*, and *T. rubrum* can be regarded as independent species despite their close similarity. Based on the sequence of the ITS ribosomal DNA barcode gene, they could be distinguished as groups. The *T. violaceum* group contained *T. violaceum var. indicum* and *T. glabrum*. The *T. soudanense* group included *T. circonvolutum, T. gourvilii var. intermedium*, and *T. gourvilii*, and the *T. rubrum* group contained type strains of *T. fischeri, T. flavum, T. fluviomuniense, T. kanei, T. pedis, T. raubitscheckii,* and *T. rodhainii*, all of which, consequently, can be regarded as proven synonyms of *T. rubrum* [18].

According to the database, *T. soudanense* was identified as such or as *T. rubrum* (African population).

Therefore, the anatomical site of isolation could be very useful as a criterion for orienting because *T. violaceum* and *T. soudanense* are prevalently found on the scalp (80.85% and 71.43% of strains from human sources, respectively), whereas *T. rubrum* is mostly found on glabrous skin (6.98% of strains from human sources) [18].

Because of the increased traveling and migration of people, the geographical origin of the strains is less reliable. With certain minor exceptions, it was found that *T. rubrum* and *T. violaceum* have a global distribution, whereas *T. soudanense* is limited to Africa [18]. However, *T. violaceum* is mainly found around the Mediterranean basin (particularly in North Africa), as well as in Central Africa, the Middle East, and Eastern Europe [10].

Based on these considerations, it appeared reasonable that our cases of tinea pedis were due to *T. soudanense* since this species is predominant in Western Africa particularly in Senegal [19, 20].

Five cases of discrepancies were found out of 32 species identification. They concerned in 2 cases, *T. rubrum* misidentified as *T. interdigitale*.

Contrary to our results, most of the discrepancies (11/14) reported by Li et al. concerned *T. mentagrophytes* misidentified as *T. interdigitale* [13]. Also, Pryce et al. observed two discordant identification results between the phenotypic identification and the ITS sequence-based identification including one case of *T. mentagrophytes* misidentified as *T. interdigitale* and another with *Chrysosporium indicum* misidentified as *T. interdigitale* [14].

In 2015, Iranian authors, using the PCR-RFLP method, noted the same discordance as we noted but with 80.8% (76/94) of *T. rubrum* misidentified as *T. interdigitale* [21]. Likewise, Tunisian authors reported this misidentification, *T. rubrum* as *T. mentagrophytes* or inversely with 16.6% (4 of 24 cases) [22]. It has to be said that Tunisian authors did not specify distinction between *T. mentagrophytes* and *T. interdigitale* in the *T. mentagrophytes* complex.

This could be justified when the identification is based only on phenotypical approach because morphologically, *T. rubrum* exhibits a spectrum of overlapping characters. This is why so many varieties or synonyms have been described in the past (cited above). Also, culture on Bromocresol purple Agar (BCP), hydrolysis of urea, and hair perforation test was included to the confirmatory test of *T. rubrum* [11].

In the current classification, the name *T. interdigitale* is reserved for exclusively anthropophilic isolates, mainly found in tinea unguium and tinea pedis cases, as opposed to zoophilic *T. mentagrophytes* isolates, which also can be found in clinical cases other than nail and foot infections [23].

This consideration is in line with our results with 88.9% (8/9) of *T. interdigitale* isolates from interdigital tinea pedis (6/8) and tinea unguium (2/8).

Taghipour et al. in 2019 noted a statistically significant difference in the ITS genotype distribution between different affected areas of fungal infection [23]. On the other hand, they found no *T. interdigitale* strains isolated from tinea capitis cases contrary to our findings with one case of *T. interdigitale* tinea capitis (MN691059).

Besides, for our strains, only urease test was performed on a urea-indole tub which was useful to distinguish *T. interdigitale* (urease test positive in 2 days) to *T. rubrum* (negative test in 2 days).

Although this study focused on the comparison of phenotypic and molecular identification of dermatophytes using DNA sequencing of the ITS regions, it would be interesting to be expanded and to include other molecular markers with high discriminating power, because a high genetic diversity could suggest a better capacity of adaptation under a selective pressure [24].

## 5. Conclusion

In definition, phenotypic methods do not seem to pose a diagnostic problem concerning identification of dermatophytes mostly for the main commonly isolated dermatophytes species in Senegal. During this study, phenotypic methods were confirmed by DNA sequencing of the ITS regions up to 90% for some species. However, it seems inadequate in case of atypical morphology or pleomorphism. Thus, molecular techniques must prevail in our laboratories to better identify for ensuring better care.

## Figures and Tables

**Table 1:** Universal ITS primers and amplified regions [12].

Noun/pair	Universal primer sequence (5′ ⟶ 3′)	Amplified regions
ITS1/ITS2	ITS1: 5′-TCCGTAGGTGAACCTGCGG-3′	3′end 18S, ITS1, and 5′ end 5, 8S
	ITS2: 5′-GCTGCGTTCTTCATCGATGC-3′	

ITS3/ITS4	ITS3: 5′-GCATCGATGAAGAACGCAGC-3′	3′ end 5, 8S, ITS2, and 5′ end 28S
	ITS4: 5′-TCCTCCGCTTATTGATATGC-3′	

ITS1/ITS4	ITS1: 5′-TCCGTAGGTGAACCTGCGG-3′	3′ end 18S, ITS1, 5, 8S, ITS2, and 5′ end 28S
	ITS4: 5′-TCCTCCGCTTATTGATATGC-3′	

**Table 2:** Details of dermatophyte strains isolated in Dakar, Senegal, with correlation and discordances between phenotypic and molecular identification by DNA sequencing of the ITS regions.

GenBank accession numbers	NO	Origin	Identification	
	*D*		Phenotypic	Molecular
MN691044	**1**	TC	*M. audouinii*	*M. audouinii*
MN691045	**2**	TC	*T. soudanense*	*T. soudanense*
MN691046	**3**	TC	*M. audouinii*	*M. audouinii*
MN691047	**4**	TC	*M. audouinii*	*M. audouinii*
MN691048	**5**	TC	*T. soudanense*	*T. soudanense*
MN691049	**6**	TC	*T. soudanense*	*T. soudanense*
MN691050	**7**	TC	*M. audouinii*	*M. audouinii*
MN691051	**8**	TC	*T. soudanense*	*T. soudanense*
MN691052	**9**	TC	*M. canis*	*M. canis*
MN691053	**10**	TC	*T. soudanense*	*T. soudanense*
MN691054	**11**	TC	*T. soudanense*	*T. soudanense*
MN691055	**12**	TC	*T. soudanense*	*T. soudanense*
MN691056	**13**	TC	*M. audouinii*	*M. audouinii*
MN691057	**14**	TC	*T. soudanense*	*T. soudanense*
MN691058^*∗*^	**15**	TC	*T. mentagrophytes*	*T. soudanense*
MN691059^*∗*^	**16**	TC	*T. soudanense*	*T. interdigitale*
MN691060	**17**	ITP	*T. interdigitale*	*T. interdigitale*
MN691061	**18**	ITP	*T. rubrum*	*T. rubrum*
MN691062	**19**	ITP	*T. interdigitale*	*T. interdigitale*
MN691063	**20**	ITP	*T. interdigitale*	*T. interdigitale*
MN691064	**21**	ITP	*T. interdigitale*	*T. interdigitale*
MN691065	**22**	ITP	*T. interdigitale*	*T. interdigitale*
MN691066	**23**	ITP	*T. interdigitale*	*T. interdigitale*
MN691067^*∗*^	**24**	ITP	*T. interdigitale*	*T. rubrum*
MN691068^*∗*^	**25**	ITP	*T. interdigitale*	*T. rubrum*
MN691069	**26**	TU	*T. soudanense*	*T. soudanense*
MN691070	**27**	TU	*T. soudanense*	*T. soudanense*
MN691071	**28**	TU	*T. interdigitale*	*T. interdigitale*
MN691072	**29**	TU	*T. soudanense*	*T. soudanense*
MN691073	**30**	TU	*T. interdigitale*	*T. interdigitale*
MN691074	**31**	TCO	*T. soudanense*	*T. soudanense*
MN691075^*∗*^	**32**	MYC	*Unidentified*	*M. audouinii*

ITP: interdigitale tinea pedis; TC: tinea capitis; TCO: tinea corporis; MYC: mycetoma; TU: tinea unguium; ^*∗*^cases of discrepancies.

## Data Availability

Data used to support the findings of this study are included within the article. However, sequences have been deposited in GenBank (https://blast.ncbi.nlm.nih.gov/Blast.cgi) with GenBank accession numbers from MN691044 to MN691075.
